# Autonomous Water Sampler for Oil Spill Response

**DOI:** 10.3390/jmse10040526

**Published:** 2022-04-11

**Authors:** Daniel Gomez-Ibanez, Amy L. Kukulya, Abhimanyu Belani, Robyn N. Conmy, Devi Sundaravadivelu, Lisa DiPinto

**Affiliations:** 1 Woods Hole Oceanographic Institution, Woods Hole, MA 02543, USA; 2 U.S. Environmental Protection Agency, Office of Research and Development, Cincinnati, OH 45268, USA; 3 Pegasus Technical Services, Inc., Cincinnati, OH 45219, USA; 4 National Oceanic and Atmospheric Administration, Silver Spring, MD 20910, USA

**Keywords:** oil spill, water sampling, autonomous underwater vehicle, autonomous survey, organic analysis, environmental impact assessment, rapid response, plume tracking

## Abstract

A newly developed water sampling system enables autonomous detection and sampling of underwater oil plumes. The Midwater Oil Sampler collects multiple 1-L samples of seawater when preset criteria are met. The sampler has a hydrocarbon-free sample path and can be configured with several modules of six glass sample bottles. In August 2019, the sampler was deployed on an autonomous underwater vehicle and captured targeted water samples in natural oil seeps offshore Santa Barbara, CA, USA.

## 1. Introduction and Background

Underwater oil spills can have long lasting impacts to ocean and coastal environments, but are challenging to observe. A newly developed autonomous underwater vehicle (AUV) water sampling system uses in situ sensing to detect dissolved oil, and then immediately capture seawater in quantities sufficient to discriminate and measure its hydrocarbon constituents while maintaining the independence and integrity of multiple samples.

This section reviews the need for midwater sample collection and previous work in autonomous water sample collection. Section 2 describes the concept and components of the Midwater Oil Sampler (MOS). Section 3 describes the qualification tests during design development, and Section 4 describes at-sea deployment of the Midwater Oil Sampler. Discussion and conclusions are given in Sections 5 and 6.

### 1.1. Obstacles to Underwater Oil Sampling

Underwater oil spill response relies on complementary observations from remote sensing, shipboard measurements, and remotely operated and autonomous vehicles, all of which have a role in understanding the flows of oil released into the ocean. However, only laboratory chemical analysis of water samples can distinguish hydrocarbon classes and accurately measure concentrations. This information is important to understanding the fate and effects of oil in the ocean.

The Deepwater Horizon disaster of 2010 made clear that an underwater oil spill is different from a surface spill, with a significant fraction of hydrocarbons retained in midwater [1], and is therefore inaccessible to traditional methods of surface water collection. However, even 12 years after Deepwater, it is challenging to access, locate, and sample a midwater oil plume [2].

Obstacles to effective sampling of a midwater oil plume include, first of all, site access, followed by the detection of the plume, and finally, the capture and shipping of water samples.

#### 1.1.1. Site Access

AUVs offer a uniquely capable platform for monitoring the extent of a midwater oil plume, which may be difficult to reach due to depth, remoteness, competing marine traffic, and hazards to human health and safety.

Towed or tethered remotely operated underwater vehicles (ROVs) and human occupied vehicles (HOVs) can reach abyssal depths and offer immediate visual feedback to operators. However, ROVs are limited to operations close to a surface vessel, and surface vessels may be unavailable or not allowed to approach disaster sites due to ongoing response efforts. Vessels that do approach may be exposed to hazards including crude oil vapor, smoke, and explosions.

AUVs, in contrast, offer over-the-horizon access. They can be launched from surface vessels beyond the disaster area, or from shore. AUVs can be mobilized quickly and work independently from surface vessels, covering a large area to unlimited depths, discovering midwater plumes that may not be connected to any visible surface expression. AUVs can be programmed to travel in a lawnmower or sawtooth pattern, creating an XY grid map or YZ section, both of which are impractical with an ROV. With each surfacing, an AUV may relay data via satellite to a land-based server for human interpretation and situational awareness. AUVs can be programmed to perform a broad scale search, first covering the search area with widely spaced profiles, and later returning to areas with above-average fluorescence detections or ‘hot-spots’ for a fine-scale survey using more focused sensors such as cameras or water collection devices. This multi-scale survey can be automated and is adaptable in real-time depending on changing mission objectives and situational awareness.

#### 1.1.2. Oil Detection

In order to sample oil released underwater, it must first be detected using remote or in situ sensors. Transported by ocean currents and its own buoyancy, the oil plume may eventually reach the surface, forming a surface expression or “slick”, which can be detected visually or with radar [3] from a ship, airplane, or satellite. However, some oil may be neutrally buoyant at depth with no obvious connection to surface features [4].

In situ fluorometers are used to locate midwater oil plumes [5]. The Seabird SeaOWL oil-in-water sensor detects fluorescent dissolved organic material (FDOM) with excitation at 370 nm and emission at 460 nm [6]. SeaOWL also measures chlorophyll fluorescence, with an excitation at 470 nm and emission at 690 nm, and backscatter at 700 nm, which can help to disambiguate dissolved oil from other sources of FDOM. The SeaOWL is used in real-time to target sample collections during an adaptive sampling mission.

#### 1.1.3. Sample Capture and Shipping

Analysis of dissolved oil samples using gas chromatography mass spectroscopy (GC-MS) can discriminate 91 well-known hydrocarbons, producing a fingerprint of the hydrocarbon source [7]. However, to deliver useful samples to a lab, the samples must be collected and transported without altering hydrocarbon constituents.

Carryover contamination occurs when oil from one location is present in samples intended to represent a different location. A surface slick may adhere to collection equipment that is deployed to deeper water, causing oil to be detected at depths where there is none. Decontamination of sampling equipment between deployments can be a challenge, since clean water may be unavailable. If a common inlet tube is used to collect samples at several locations, residual oil from the inlet may be distributed among all samples. To minimize carryover contamination and simplify site decontamination, single-use disposable sampling equipment should be used when feasible [8].

If water samples are exposed to heat or sunlight, volatile hydrocarbons may be lost by evaporation. If water is collected in one container and then transferred to another, sampled oil may remain stuck to the first bottle or transfer tubing, reducing the measured concentration. When feasible, a water sampling system should avoid bottle-to-bottle transfers and facilitate offload to cold storage immediately after collection.

Containers used to hold water samples may affect the measured hydrocarbon concentrations [9]. If samples are temporarily stored in plastic bottles, hydrocarbons can migrate from the bottle to the water sample or vice versa. Volatile hydrocarbons may diffuse through a plastic bottle and escape before analysis. To minimize these effects, typical sampling protocols specify only glass bottles for oil sampling [8,10].

### 1.2. Existing Sampling Methods

Near-surface oil can be collected manually by direct dip or peristaltic pump [8], but these methods are not feasible beyond a few meters of depth.

Deeper samples can be collected remotely using ROV-operated Niskin bottles, small-diameter tubing [11], syringes [12], or gas-tight bottles [13]. However, these remote capture devices are not suitable for AUV integration due to large size and weight.

A few water samplers have been integrated with long range AUVs. The Aqualab sampler [14,15] used a multiport valve and 49 ethylene vinyl acetate (EVA) pouches to collect 200 mL water samples beneath fjords in East Greenland in 2004 for isotope ratio analysis, demonstrating the utility of using autonomous vehicles to retrieve samples from otherwise inaccessible sites. The Suspended Particulate Rosette (SUPR) family of samplers [16] also uses a multiport valve, but allows for different bottle sizes and materials to be used for compatibility with various analytes. The Gulper [17,18] uses polymethyl methacrylate (PMMA) plastic sample cylinders and silicone o-rings, materials selected for consistent phytoplankton growth in primary productivity studies. Gulper was deployed to collect water samples during the Deepwater Horizon oil spill response, although sampling did not occur due to a flooded controller housing [4].

## 2. Midwater Oil Sampler Design, Materials, and Methods

This section describes the Midwater Oil Sampler and the other components of a complete autonomous water sampling system including the AUV host platform, in situ sensor suite, the Midwater Oil Sampler module, its operation sequence, and mission programming. These parts work together to efficiently collect targeted water samples.

### 2.1. Host Platform

A REMUS 600 AUV [19] was chosen as a host platform for the Midwater Oil Sampler because it is large enough to comfortably accommodate several 1-L bottles, along with associated sensors, within its 32 cm diameter. The REMUS 600 operates to a 600 m depth, with 3 m length, 300 kg mass, and expandable 5 kWh battery energy. Its aft section implements core vehicle control, propulsion, energy storage, and communication functions, while its forward section is a modular payload that can accommodate a variety of mission-specific sensors using one of the supported payload electrical interfaces. The REMUS 600 is a member the REMUS family of AUVs, which includes the smaller REMUS 100 and the larger REMUS 6000 autonomous underwater vehicles, developed at the Woods Hole Oceanographic Institution and commercialized by Hydroid Inc. (Pocasset, MA, USA). The Midwater Oil Sampler requires only DC power and RS-232 communication links to the host, so it can also be used with other similar vehicles.

### 2.2. Integrated In Situ Sensors

For oil spill response, the REMUS 600 AUV was configured with several in situ sensors along with the newly developed Midwater Oil Sampler. The sensor suite included a Licor LI-192 photosynthetically active radiation (PAR) sensor, Seabird Sea-OWL fluorometer, Anderaa 4831F optode, Seascan Holocam, and a GoPro Hero 3 video camera. Only the fluorometer was used for real-time triggering of water samples; other sensor data were analyzed post-mission to serve as converging lines of evidence to confirm the presence of oil.

### 2.3. Sampler Design and Fabrication

The Midwater Oil Sampler was designed to minimize the contamination of samples through the use of compatible materials and an optimized flow path. Both are described in this section, along with the fabrication and assembly of the Midwater Oil Sampler.

#### 2.3.1. Material Selection

Glass sample bottles were used to avoid hydrocarbon contamination that occurs with plastic sample containers [9]. Single-use pre-cleaned wide-mouth amber bottles are readily available from several vendors. Bottles were certified by the vendors to meet the U.S. Environmental Protection Agency (EPA) standards [20], with negligible amounts of semi-volatile organics and other contaminants. Glass bottles may optionally be baked in a muffle furnace for decontamination and reuse. The sample volume of 1 L was chosen to maintain typical detection limits while still allowing several samples to fit in an AUV payload. One liter samples are consistent with the water sampling procedures developed in response to the Deepwater Horizon disaster [8]. After recovery of the host AUV, sample bottles can be quickly removed from the sampling system, capped, and shipped to a laboratory.

The only materials in the sample path other than the glass bottle were 316 stainless steel and fluoropolymers (PFPE, FKM, and PTFE) to minimize hydrocarbon contamination.

#### 2.3.2. Flow Path

**Figure 1.**
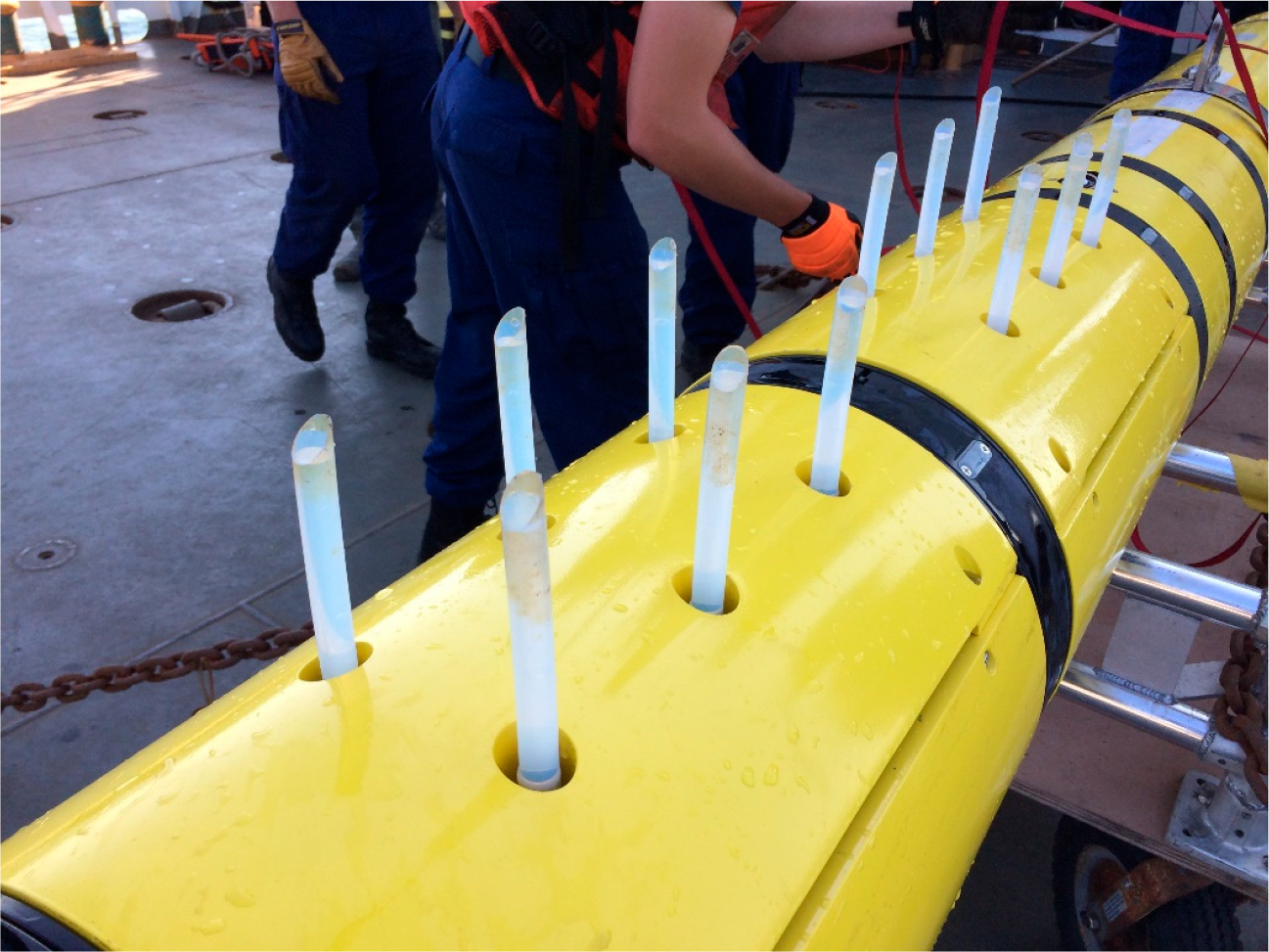
REMUS 600 AUV integrated with the Midwater Oil Sampler.

The configuration of the water flow path was designed to minimize contamination. Sample inlets for each bottle were independent to minimize carryover contamination. Water entered each bottle through a 12 mm diameter by a 15 cm long upward-facing PTFE inlet extension tube, which extended beyond the vehicle envelope to avoid collecting water that has been in contact with the AUV skin. Inlet tubes were replaced after each mission to avoid carryover contamination between missions and remove residue from surface slicks, which is clearly visible post-mission in Figure 1. Past the inlet, water flowed through a check valve and into a sample bottle via the bottle adapter. Check valves (Swagelok SS-4CP6-1/3-SC11) trap water in the bottle and prevent water exchange with the environment when the pump is not operating. The check valve and bottle adapter were made of 316 stainless steel with fluoropolymer o-rings lubricated with Krytox fluoropolymer grease. Bottle adapter assembly included a stainless-steel fill-tube inside the bottle to promote complete water exchange. A second check valve was connected at the outlet port of the bottle adapter.

Because the pumps and other tube fittings were located downstream of the sample bottle, beyond a check valve, they were not in the sample path and did not need to be completely free of hydrocarbon contaminants. The outlet check valve was followed by a brass 90° elbow, followed by a silicone 90° elbow, which was connected to a pump. Each bottle had an independent water pump to pull pre-fill water out of the bottle during sampling. Submersible impeller pumps (Shenzhen Century Zhongke Technology Co., Shenzhen, China, model DC40-1250) were potted without voids for pressure tolerance.

#### 2.3.3. Fabrication and Assembly

The Midwater Oil Sampler uses off-the-shelf parts, rapid prototyping, and 3D-printing to support efficient sampler fabrication and assembly.

**Figure 2.**
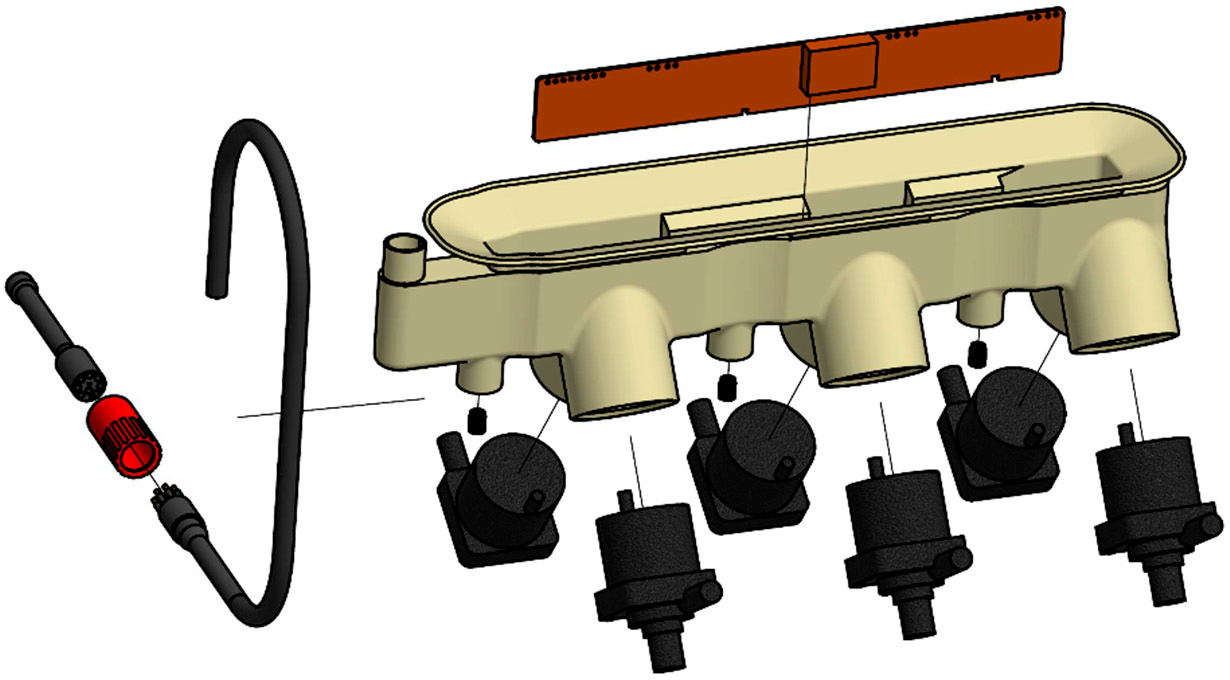
Exploded view of the junction box sub-assembly. Parts are, clockwise, control PCBA, junction box shell, six pumps, and uplink umbilical cable.

A junction box subassembly (Figure 2) housed six pumps and supporting electronics, which were sealed together in cast polyurethane within a thin plastic shell. The junction box shell was formed by fused deposition modeling (FDM) of an acrylonitrile styrene acrylate (ASA) filament. Before potting, pump housings were sandblasted to improve adhesion to polyurethane, and then inserted into circular openings in the shell. Inside the shell, uplink cable and motor wires were soldered to a control printed circuit board assembly (PCBA). The control PCBA accepts a power supply of 18–36 V from the host AUV and communicates with the host via RS-232. The control PCBA powers each pump for a specified time. The PCBA also provides diagnostic information including power consumed by each pump, temperature, and insulation resistance. Polyurethane (3M Scotchcast 2131) was poured into the shell, degassed under vacuum, and cured overnight.

**Figure 3.**
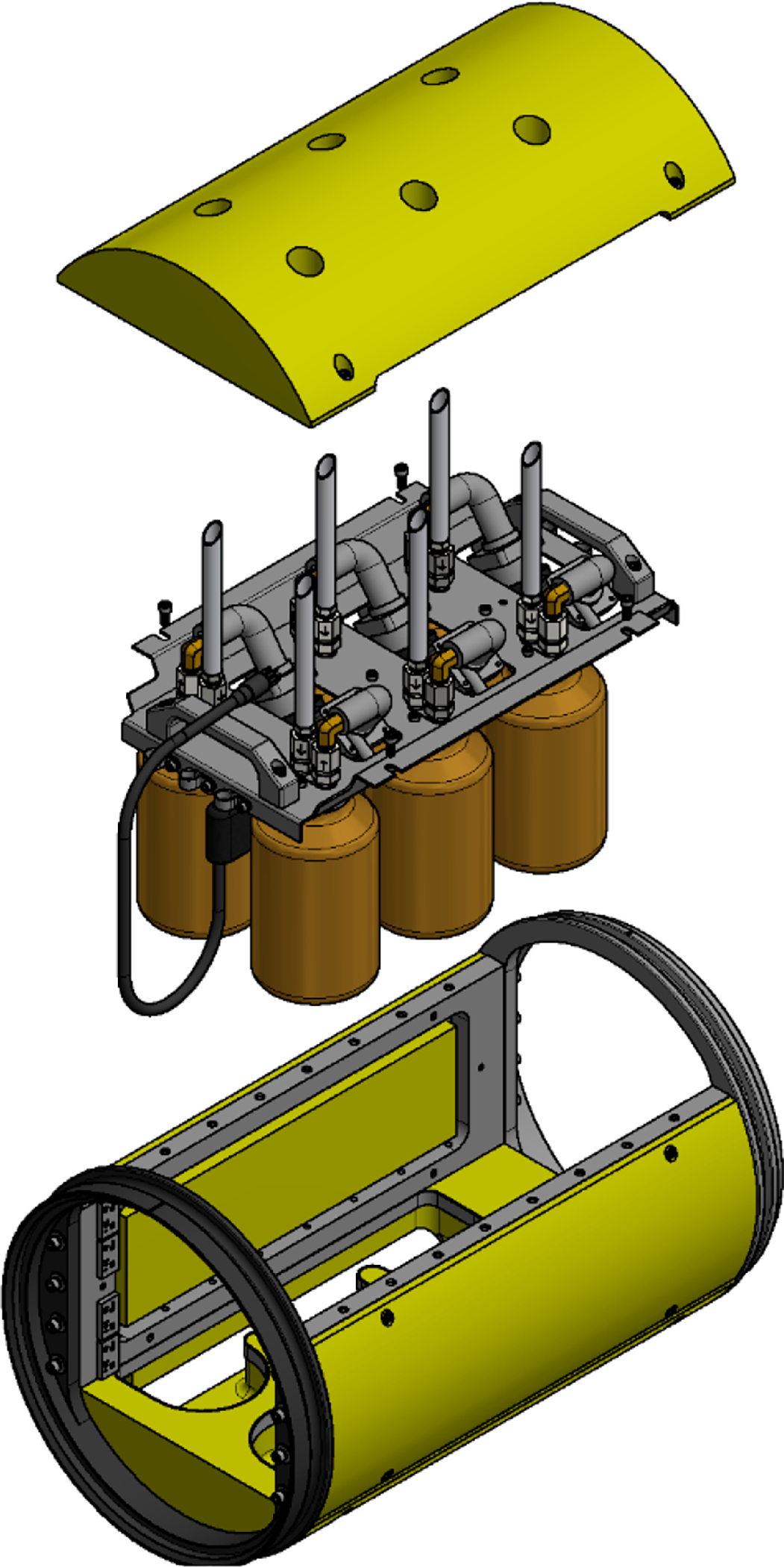
Exploded view of the Midwater Oil Sampler module. From top to bottom are the top foam block, sampler module, and sampler frame.

The junction box subassembly and six bottle-adapter assemblies were all attached to an anodized aluminum plate, with handles to facilitate installation of the 6-bottle module into the host AUV (Figure 3). Each six-bottle sampling module was placed in a rigid aluminum frame attached to the host AUV using 32 cm diameter ring joints. Yellow-painted syntactic foam blocks were attached to the frame, surrounding the sample bottles. These foam blocks protect the glass bottles, streamline the vehicle, and offset the weight of the sampler and frame. More than one sampler frame may be mounted on an AUV at the same time, allowing for 6, 12, or 18 samples to be collected during a single REMUS 600 mission.

### 2.4. Operation Sequence

**Figure 4.**
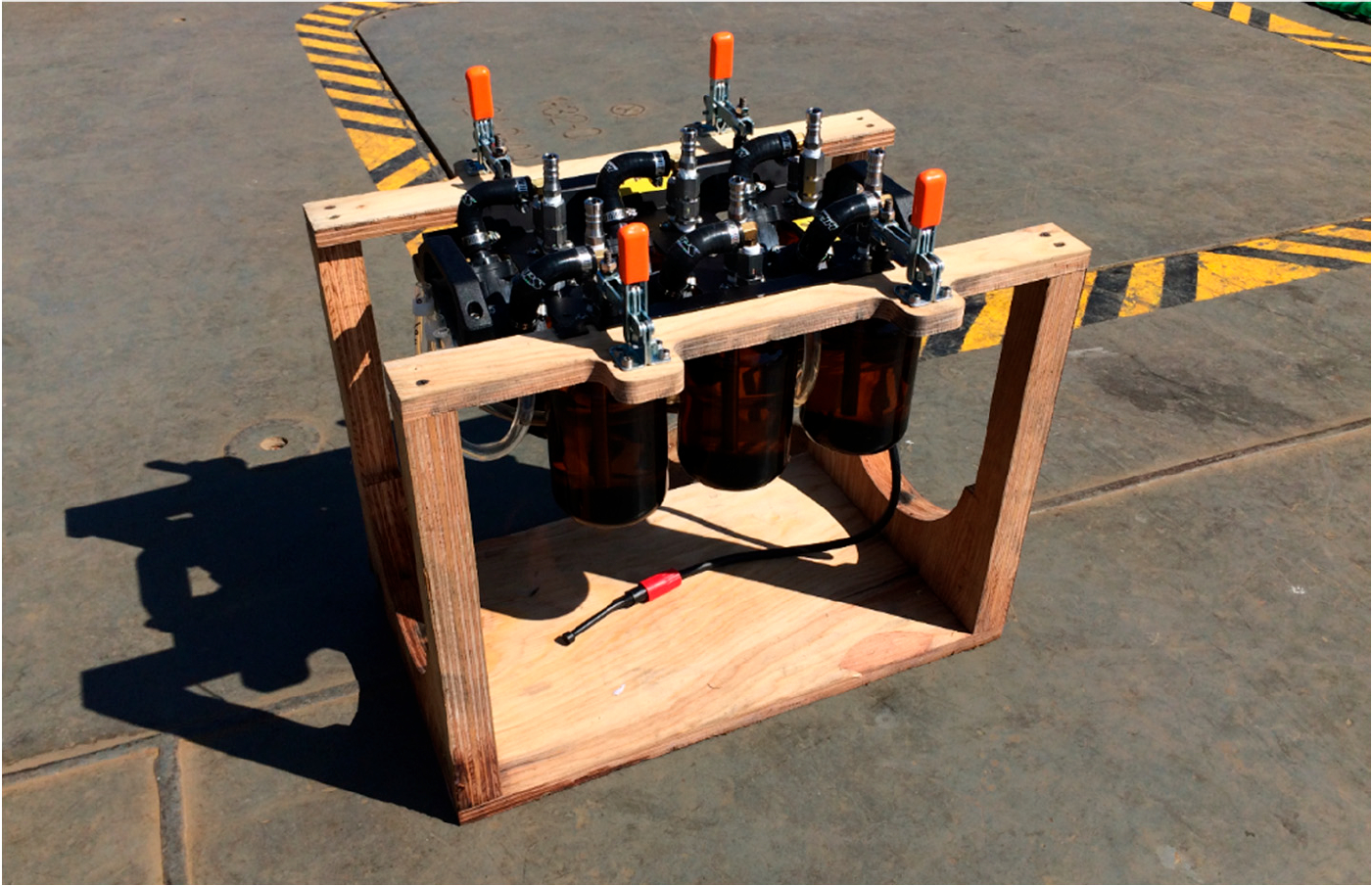
One sampler module in its plywood service cradle.

For convenient bottle preparation and fast access to samples, modules of six bottles are easily removable from the AUV. The following steps were used to prepare and operate the Midwater Oil Sampler:

1. Clean bottles are installed by screwing them in from below. The sampler’s plywood service cradle provides clearance below the sample bottles (Figure 4).
2. Distilled water is pumped into each bottle by temporarily connecting a flexible tube from a distilled water supply to each bottle inlet.
3. One or more modules are loaded into the host AUV. The sampler umbilical cable is mated, and the top foam block fastened over the top of the sampler. New inlet extensions are attached to each inlet barb. The vehicle is now ready to launch.
4. During an underwater mission, when a pump is activated by the host AUV, ambient seawater flows into one bottle, passing through the inlet extension, inlet check valve, and fill tube.
5. After the host AUV is recovered, the top foam block is removed, and the umbilical cable is disconnected. The sampler is removed and placed in its service cradle.
6. Sample bottles, attached to the sampler only by their threaded necks, are unscrewed and capped with PTFE-lined screw caps. No bottle-to-bottle water transfer is necessary.
7. Bottles are labeled and placed in coolers for transport to the laboratory.

### 2.5. Mission Programming and Sample Triggering

The host REMUS 600 AUV control system is responsible for dynamically positioning the vehicle within an oil plume and triggering the sampler to fill a bottle. The AUV operator specifies intent in the form of a mission script, composed of several sequential objectives. Sampling can be accomplished with either of two different mission objectives developed for plume sampling. To sample at a specified position, a *Point and Gulp* objective may be used. To sample when certain fluorometer (FDOM) criteria are met, an *Adaptive Sampling* objective may be used. The *Point and Gulp* objective is used when desired sampling locations are known in advance, based on previous AUV missions or other observations. During the *Point and Gulp* objective, the vehicle transits to a specified latitude, longitude, and depth, and then navigates, at constant depth, in a 20 m radius circle around the specified point, at 2 knots speed, through two revolutions. This circle pattern allows enough time for the sampling pump to run for 90 s, exchanging pre-fill water with ambient seawater.

The *Adaptive Sampling* objective is intended to provide additional observations when elevated fluorescence is detected and may optionally be configured to take a water sample. Typically, an AUV survey consists of a series of grid lines covering an area of interest. The *Adaptive Sampling* objective allows the survey path to adapt to observations. When the triggering criteria are met, the vehicle navigates in a spiral around the point of highest FDOM, gathering additional observations, before resuming the previously specified wide-area grid survey. The *Adaptive Sampling* objective allows the triggering criteria for water sampling to be set independently from the triggering criteria for additional survey passes.

Triggering criteria include “thresh_min”, which specifies a minimum FDOM count, and “thresh_inflate” (i.e., how much higher the background must be for the FDOM sensor measurement to trigger optional observation or sampling). For example, a 10% increase above background fluorescence could trigger additional observations, while a 20% increase could trigger a water sample. Water sampling occurs only after additional observations are completed, and the water sampling circle is centered at the point of highest FDOM.

## 3. Design Verification

Several important functions of the Midwater Oil Sampler were tested before its first deployment in a midwater oil plume. Verification tests are outlined below.

### 3.1. Pressure Tolerance

Pumps were qualified for use at ambient pressure by placing one pump in a hydrostatic pressure test chamber filled with mineral oil, with electrical penetrations to supply 12 V DC power to the pump inside. The chamber was pressurized to 69 MPa, equivalent to 7000 m depth, with the pump operating. The pump was run for 24 h while pressurized, with no change in power consumption. Pump flow rate was found to be unchanged after the test.

After the assembly of pumps into the junction box, each complete multi-pump assembly was placed underwater and electrical resistance from umbilical power ground pin to seawater was measured with a multimeter. No ground faults were detected in any of the assembled junction boxes.

### 3.2. Flow Rate

To measure the pump flow rate, each complete sampler was filled with fresh water, and the inlet tube was immersed in a bucket of water. A single pump was activated continuously. Water exiting the pump was collected in empty 1 L bottles. With each sampler tested, bottles filled in 20–30 s. Variability was mainly due to air trapped in the impeller housing, something that occurs during bench testing, but not when submerged. In this application with check valves and other restrictions, flow rate was conservatively estimated to be greater than 2 L per minute. To ensure complete displacement of pre-fill water by ambient (sample) seawater, pumps were run for 90 s, enough for at least three water changes.

### 3.3. Exchange Fraction

To promote rapid and complete exchange of pre-fill with ambient seawater, an inlet tube extended down from the bottle adapter into the bottle to guide newly introduced seawater to the bottom of the sample bottle, while displaced water exits out of the top. Tests using dye-colored water to track the exchange of water in the bottle confirmed that this tube promotes a more complete exchange of water for each liter pumped. A density gradient between buoyant distilled pre-fill and heavier saline sample water also helps keep the newly introduced seawater below and separate from the less dense pre-fill, so that the bottle is filled from bottom to top with little mixing. To verify the exchange of water, a sampler was pre-filled with distilled water, placed in seawater, and each pump was commanded to pump for 90 s. After pumping, the salinity in the bottle was within ±3% of ambient seawater, indicating that little of the original pre-fill water remained in the bottle after sampling.

### 3.4. Leak Testing

The water sampler secured water in the sample bottle by means of two check valves. These valves were sealed closed by springs when the pumps were powered off. To test the integrity of the seals, six bottles were pre-filled with fresh tap water, measuring less than 1 ppt salinity. The entire sampler was suspended in seawater overnight. After removing the bottles, salinity was measured again and remained less than 1 ppt.

Performance of the check valves can also be verified during operation of the sampler by using distilled water for pre-fill and reserving a “trip blank”, a bottle that is never pumped. By measuring the salinity of the trip blank, the integrity of bottle seals can be confirmed during actual sampling operations.

## 4. At-Sea Testing Results

In August 2019, a multi-agency cooperative exercise located, mapped, and sampled naturally occurring oil seeps near Santa Barbara, California, USA. The exercise simulated a rapid response to an oil spill, and employed U.S. Coast Guard buoy tender George Cobb, several underwater vehicles, an aerial drone, and satellite remote sensing. Participants from several organizations gained familiarity with the AUV operations, developed processes to deliver data products in near real-time to the U.S. National Oceanic and Atmospheric Administration’s Environmental Response Management Application (ERMA) geodatabase, and demonstrated the integration of AUV operations as part of a coordinated oil spill response [21].

This section describes the autonomous survey missions, water sampler operation, and sample analysis.

### 4.1. Autonomous Survey Summary

A REMUS 600 AUV, with in situ sensing and Midwater Oil Sampler payloads, conducted gridded surveys of areas known to host underwater oil seeps. The REMUS 600 executed 19 missions in five days, covering a total distance of 126 km. Total survey time was 23 h and 55 min, while individual missions lasted between 0.5 and 3 h.

Discharges from several natural gas and oil seeps were variable during the plume mapping exercise. To locate and sample active oil plumes, it was valuable to have multiple aerial and underwater vehicles active simultaneously, providing real-time situational awareness, which was used to plan short 1 to 2 h underwater sampling missions based on the most recent observations. Thirteen of 19 missions did not detect FDOM above the threshold for sampling. Over five days, the AUV collected a total of 13 seawater samples.

### 4.2. Water Sampler Operation

In preparation for the oil plume mapping exercise, eight water sampler modules were staged on the Cobb. Distilled water was purchased on shore and loaded on to the Cobb in 20 L polycarbonate carboys. This water was used to pre-fill empty sample bottles before loading the sampler modules into the REMUS 600 AUV. Before each day of the AUV operations, a total of twelve 1 L bottles were loaded into two water sample modules.

At the end of each day’s operations, newly filled bottles were removed from the samplers. Two 40 mL subsamples were poured from each sample bottle into glass vials required for benzene, toluene, ethylbenzene, and xylene (BTEX) analysis. The original sample bottles with remaining water were capped and labelled with the date, sampler serial number, and pump number. Bottles were packed in ice for overnight shipping to a lab for analysis.

After removing the sample bottles, the samplers were decontaminated by first scrubbing the sampler in a tub of Sunshine Makers Simple Green All-Purpose Cleaner diluted 10:1 with water, and then pumping a solution of 10:1 diluted Simple Green from a reservoir through each inlet and pump channel, with a temporary bottle installed, followed by a freshwater rinse.

### 4.3. Laboratory Water Analysis

A total of 19 water sample bottles were sent to a U.S. EPA lab in Cincinnati and analyzed within 14 days of collection. Thirteen bottles contained seawater samples and five bottles were blanks, filled with distilled water, and carried onboard the AUV during one or more missions but never pumped. These bottles were known as trip blanks. One bottle was pre-filled with distilled water, but was never loaded onto a sampler, never placed into the ocean, and known as a DI (deionized water) blank.

In the laboratory, salinity was measured using a portable refractometer at 22 °C. All thirteen seawater samples exhibited salinity above 30 ppt, providing confidence that the pumps effectively exchanged the blank freshwater with ambient seawater.

The 40 mL subsamples were used for volatile organic compound (VOC) analysis using Method 524.3 [22] modified to use dynamic headspace extraction instead of purge and trap.

Other hydrocarbons were extracted from the 1 L bottles with dichloromethane and concentrated to 1 mL following Method 3500C [23]. Alkanes and polycyclic aromatic hydrocarbon (PAH) concentrations were quantified by gas chromatography/mass spectrometry (GC/MS) following Method 8270D [7]. Concentrations of individual alkanes and PAHs above the reporting limits were summed to compute total alkane and PAH concentrations.

The water sample analysis results are listed in Table A1.

## 5. Discussion

**Figure 5.**
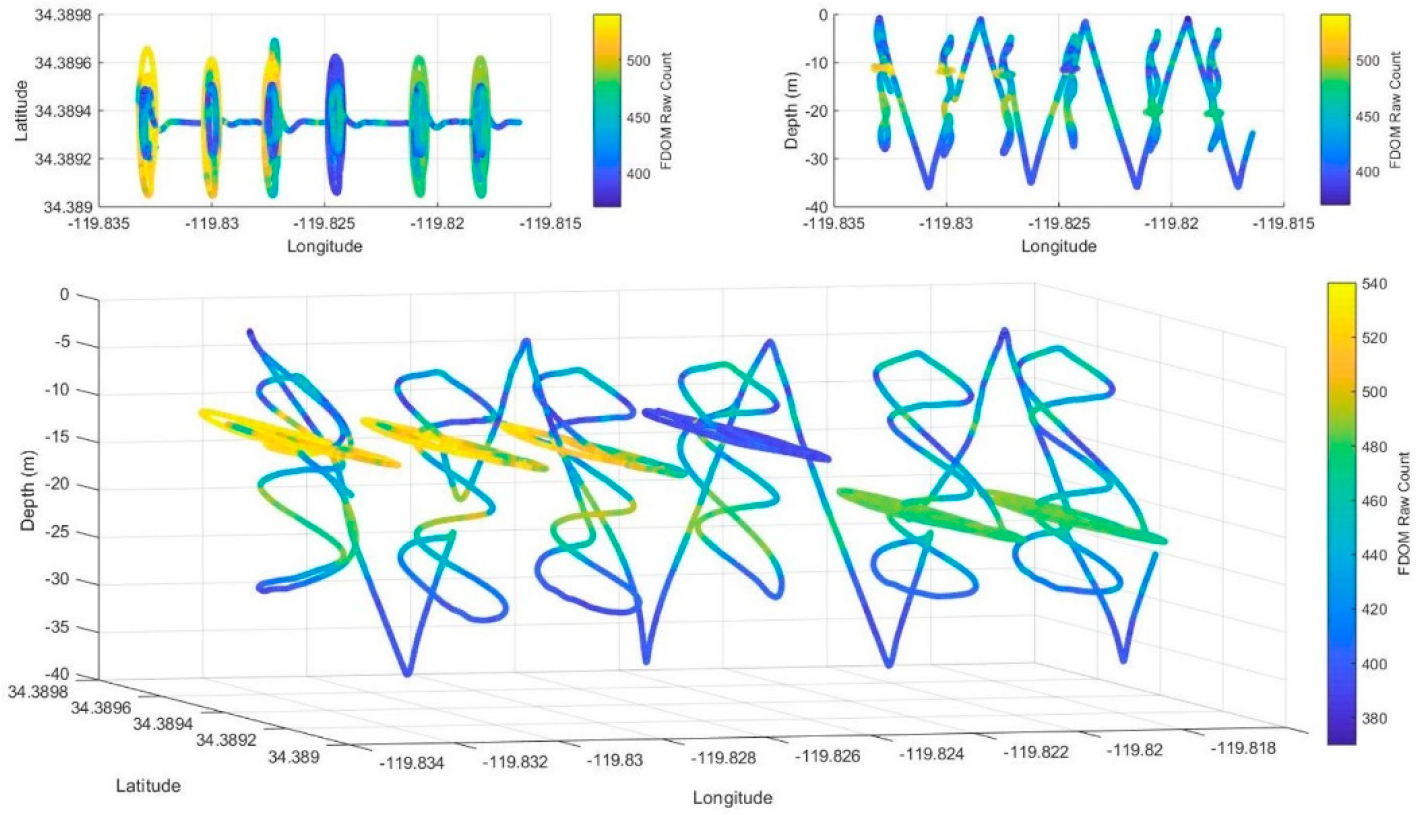
Vehicle track during REMUS 600 Mission 7, 27 August 2019. The color of the track indicates the measured fluorescence.

In the coastal waters of Santa Barbara, there are limited freshwater sources of fluorescent natural organic matter entering the ocean. Thus, salinity is high and background FDOM fluorescence is predictably stable at ~2 ppb quinine sulfate equivalent (QSE) with slightly higher concentrations in the surface waters. Deviations from these concentrations indicate sources of new fluorescent material such as petroleum hydrocarbons. Mission 7, completed on 27 August 2019, provides an example of a successful *Adaptive Sampling* objective. At six locations along a transect, as shown in Figure 5, the AUV encountered FDOM significantly above the background. After a helical search to locate the depth with highest FDOM, the AUV then collected a water sample at each location. The first four samples were collected at a 11–13 m depth and two more were collected 20 m below the surface.

Vertical depth profiles showed that SeaOWL FDOM and backscatter maxima occurred between 10 and 15 m for waters in Mission 7, which was not coincident with the chlorophyll maximum at 7 m depth. This suggests that the elevated FDOM and backscatter observed at these locations were not from biological activity. The presence of oil was also supported by the AUV’s holographic camera, which detected oil droplets at these locations [20], even though elevated levels of hydrocarbons were not detected in seawater samples from Mission 7.

Alkanes, PAHs, and BTEX measured by GC/MS in all seawater samples collected during the spill response exercise were uniformly low. Concentrations of individual hydrocarbons in the seawater samples were similar to concentrations found in trip blanks, the DI blank, and lab reagent blank. These measurements were consistent with uncontaminated samples from a site with very little oil present.

Sampler 4 was reused on 29 August after Simple Green site decontamination on 27 August. Samples and blank offloaded from the site-decontaminated sampler showed no increase in hydrocarbons compared to other samples and blanks, suggesting that Simple Green site decontamination does not introduce additional hydrocarbons into the samples.

The results of sea testing and sample analysis support the absence of contamination from the Midwater Oil Sampler itself. However, further trials in midwater plumes with higher concentrations of oil are necessary to confirm the intended operation.

## 6. Conclusions

AUV-based oil sampling complements surface- and ROV-based sampling methods. AUV fluorometer surveys alone provide situational awareness during an underwater oil spill. With the addition of a water sampler to the AUV sensor payload, spill investigations can return an accurate inventory of oil constituents in a shorter time and with less effort compared to other midwater sampling methods.

The Midwater Oil Sampler was designed to minimize contamination and expedite the handling of seawater samples. The sampler was integrated with an AUV and deployed in open-ocean natural oil and gas seeps near Santa Barbara, USA. By collecting targeted seawater samples only when oil was detected via fluorescence, the time and expense of analysis was incurred only when samples contained valuable information about the oil plume.

## Data Availability

The data presented in this study are available in article.

## Appendix A

Results of the sample water chemical analysis are included in Table A1.

Bottle ID number 190826-01 was the deionized water (DI) blank, which was filled from the Santa Barbara distilled water supply but never loaded into a sampler. Bottles 190827-006-005, 190827-006-006, and 190829-004-005 were trip blanks; these were loaded into a sampling module and travelled with the AUV on several missions before being offloaded and analyzed with the other samples.

**Table A1.** Results of water hydrocarbon analysis.

Collection Date	Sample Identifier	Collection Condition	Sampler/Bottle	Depth (m)	ΣBTEX (ng/L)	ΣAlkanes (μg/L)	ΣPAHs (μg/L)
26 August 2019	190826–01	DI Blank	n/a	n/a	385.53	1.11	0.32
27 August 2019	190827–004-001	Mission 5b	4/1	11.2	255.91	1.22	0.29
27 August 2019	190827–004-002	Mission 6	4/2	9.7	192.80	1.36	0.52
27 August 2019	190827–004-003	Mission 6	4/3	14.1	223.89	1.33	0.35
27 August 2019	190827–004-004	Mission 7	4/4	11.1	92.36	1.25	0.21
27 August 2019	190827–004-005	Mission 7	4/5	11.8	96.65	1.93	0.33
27 August 2019	190827–004-006	Mission 7	4/6	12.5	147.74	2.49	0.26
27 August 2019	190827–006-001	Mission 7	6/1	11.4	45.33	2.49	0.23
27 August 2019	190827–006-002	Mission 7	6/2	20.2	147.86	2.01	0.26
27 August 2019	190827–006-003	Mission 7	6/3	20.6	123.76	1.90	0.16
27 August 2019	190827–006-004	Mission 8	6/4	13.8	136.85	0.66	0.14
27 August 2019	190827–006-005	Trip Blank	6/5	n/a	259.31	2.46	0.33
27 August 2019	190827–006-006	Trip Blank	6/6	n/a	243.27	0.98	0.39
29 August 2019	190829–004-001	Mission 16	4/1	9.0	176.36	0.70	0.20
29 August 2019	190829–004-003	Mission 17	4/3	6.2	222.97	0.77	0.28
29 August 2019	190829–004-005	Trip Blank	4/5	n/a	256.20	1.28	0.28
29 August 2019	190829–011-001	Mission 18	11/1	11.4	163.93	0.85	0.15
6 September 2019	190906-LRB	Lab Reagent Blank	n/a	n/a	32.96	0.53	0.33
